# Bioinformatics-Based Identification of Tumor Microenvironment-Related Prognostic Genes in Pancreatic Cancer

**DOI:** 10.3389/fgene.2021.632803

**Published:** 2021-06-30

**Authors:** Shaojie Chen, Feifei Huang, Shangxiang Chen, Yinting Chen, Jiajia Li, Yaqing Li, Guoda Lian, Kaihong Huang

**Affiliations:** ^1^Guangdong Provincial Key Laboratory of Malignant Tumor Epigenetics and Gene Regulation, Sun Yat-sen Memorial Hospital, Sun Yat-sen University, Guangzhou, China; ^2^Department of Gastroenterology, Sun Yat-sen Memorial Hospital, Sun Yat-sen University, Guangzhou, China; ^3^Department of Cardiology, Sun Yat-sen Memorial Hospital, Sun Yat-sen University, Guangzhou, China; ^4^Department of Gastrointestinal Surgery, Sun Yat-sen Memorial Hospital, Sun Yat-sen University, Guangzhou, China; ^5^Department of Nephrology, Sun Yat-sen Memorial Hospital, Sun Yat-sen University, Guangzhou, China

**Keywords:** pancreatic cancer, immune microenvironment, bioinformatics analysis, CXCL10 (IP-10), TCGA

## Abstract

**Objective:**

Growing evidence has highlighted that the immune and stromal cells that infiltrate in pancreatic cancer microenvironment significantly influence tumor progression. However, reliable microenvironment-related prognostic gene signatures are yet to be established. The present study aimed to elucidate tumor microenvironment-related prognostic genes in pancreatic cancer.

**Methods:**

We applied the ESTIMATE algorithm to categorize patients with pancreatic cancer from TCGA dataset into high and low immune/stromal score groups and determined their differentially expressed genes. Then, univariate and LASSO Cox regression was performed to identify overall survival-related differentially expressed genes (DEGs). And multivariate Cox regression analysis was used to screen independent prognostic genes and construct a risk score model. Finally, the performance of the risk score model was evaluated by Kaplan-Meier curve, time-dependent receiver operating characteristic and Harrell’s concordance index.

**Results:**

The overall survival analysis demonstrated that high immune/stromal score groups were closely associated with poor prognosis. The multivariate Cox regression analysis indicated that the signatures of four genes, including TRPC7, CXCL10, CUX2, and COL2A1, were independent prognostic factors. Subsequently, the risk prediction model constructed by those genes was superior to AJCC staging as evaluated by time-dependent receiver operating characteristic and Harrell’s concordance index, and both KRAS and TP53 mutations were closely associated with high risk scores. In addition, CXCL10 was predominantly expressed by tumor associated macrophages and its receptor CXCR3 was highly expressed in T cells at the single-cell level.

**Conclusions:**

This study comprehensively investigated the tumor microenvironment and verified immune/stromal-related biomarkers for pancreatic cancer.

## Introduction

Pancreatic cancer is one of the most lethal solid tumors because of the lack of early diagnosis and rapid progression that lead to poor prognosis (Kamisawa et al., 2016). It has been ranked as the third leading cause of cancer-related death in the United States with a 5-year survival rate of < 10% (Siegel et al., 2018). In the past several decades, great efforts have been made to investigate the molecular pathogenesis of pancreatic cancer; however, the advances made in the diagnosis and therapy of pancreatic cancer have still not significantly improved patient outcome (Neoptolemos et al., 2018).

One of the prominent features of pancreatic cancer is the intense desmoplastic stroma reaction, which has been considered to be an important reason for tumor progression and chemoresistance by remodeling the unique tumor microenvironment (TME) (Feig et al., 2012). TME consists of immune cells, stromal cells, endothelial cells, numerous cytokines and chemokines, and extracellular matrix molecules, which play a key role in regulating both tumorigenesis and development of cancer as well as treatment responses (Hinshaw and Shevde, 2019). Activated non-tumor cells, especially pancreatic stellate cells, produce much extracellular matrix proteins to create dense interstitial pressure that causes unavailability of nutrients and drugs (Sun et al., 2018; Thomas and Radhakrishnan, 2019). Moreover, TME may undergo dynamic changes during tumor progression depending on the interactions of tumor cells and interstitial cells. Therefore, it is critical to understand the molecular composition and function of TME to predict the prognosis of patients with pancreatic cancer.

Infiltrating immune and stromal cells are fundamental elements in TME, and increasing evidence suggest that they are closely related to tumor growth and metastasis, tumor recurrence and chemoresistance, and immune evasion (Hinshaw and Shevde, 2019). We have found that tumor-associated macrophages can promote perineural invasion and distant metastasis in pancreatic cancer (Zeng et al., 2014; Chen et al., 2018; Huang et al., 2018). Pancreatic stellate cells are also reported to play a critical role in the development and maintenance of an immunosuppressive microenvironment in pancreatic cancer (Sun et al., 2018). Hence, the investigation of the infiltrating immune and stromal cell-related gene signatures could provide novel insights and useful prognostic factors for pancreatic cancer. Estimation of STromal and Immune cells in Malignant Tumor tissues using Expression data (ESTIMATE) is an algorithm designed to evaluate the immune and stromal scores to predict both the infiltrated immune/stromal cells and tumor purity based on single sample Gene Set Enrichment Analysis (GSEA) (Yoshihara et al., 2013), and the gene list of immune and stromal signatures was shown in Supplementary Data 1. Since its development, the algorithm has been applied to various neoplasms and has shown success as a novel prognostic indicator (Wang et al., 2019; Yan et al., 2019; Chen et al., 2020).

Recently, Pu et al. (2019) used the ESTIMATE algorithm for the prognosis of pancreatic cancer, but no significant difference was found between the immune/stromal scores and overall survival. In our present study, to obtain more insights into the immune/stromal-related prognostic genes, we used the X-tile software to derive the best cutoff value based on overall survival. Independent prognostic genes of overall survival were screened by LASSO and multivariate Cox regression survival analysis, and a prognostic risk score model combining the prognostic gene signature and clinical prognostic features was established. In conclusion, our prognostic risk score model may contribute to accurately predict the overall survival of patients with pancreatic cancer.

## Materials and Methods

### Database

The RNA-seq data of pancreatic cancer from The Cancer Genome Atlas (TCGA) was downloaded from the UCSC Xena platform^1^ (Vivian et al., 2017). Clinicopathological information, including gender, age, differentiation grade, TNM stage, and survival data were also retrieved from the platform; patients who lacked complete information were excluded. In total, expression profiles of 178 pancreatic cancer samples were evaluated by the ESTIMATE algorithm to calculate immune scores and stromal scores^2^ (Yoshihara et al., 2013). The gene mutation data of TP53, KRAS, CDKN2A, and SMAD4 were obtained from the cBioPortal website^3^ (Gao et al., 2013).

### Identification of Differentially Expressed Genes

All patients with pancreatic cancer were classified into low and high score groups according to their immune/stromal scores determined by X-tile software, which takes the cutoff value based on overall survival (Camp et al., 2004). DEGs were then determined using the R package edgeR. In our study, genes with a |Log_2_FC (fold change)| > 2.5 and FDR adjusted *p*-value < 0.001 were defined as DEGs. The intersected DEGs were then screened among the immune and stromal score groups by Venn 2.1^4^.

### Functional Enrichment Analyses

Gene Ontology (GO) and Kyoto Encyclopedia of Genes and Genomes (KEGG) enrichment analyses of the intersection genes were performed with the R package clusterProfiler (Yu et al., 2012), and a FDR adjusted *p*-value < 0.05 was used as the cutoff value. The top 10 enriched GO terms and enrichment pathways of the co-underexpressed and co-overexpressed DEGs were ranked by FDR adjusted *p*-value. GSEA between the high and low immune/stromal score groups was performed in javaGSEA v. 4.0 based on “H: hallmark gene sets” from the Molecular Signatures Database (Subramanian et al., 2005). |NES| ≥ 2 and FDR adjusted *p*-value < 0.01 were considered to be statistically significant.

### Identification of Survival-Related DEGs and Construction of a Risk Stratification Model

The univariate Cox regression analysis was used to investigate the association between the intersected DEGs and overall survival of patients. DEGs with *p*-values < 0.001 were considered as candidate prognostic genes to build the risk stratification model. The LASSO algorithm was then applied to identify candidate genes by the R package glmnet with the number of lambda = 1,000 (Friedman et al., 2010). Lambda.min is the threshold value that yields minimum mean cross-validated error. Genes with the highest lambda values were chosen for performing the multivariate Cox regression analysis. DEGs with *p*-value < 0.05 were used to construct the model of prognostic signature, and the risk score (RS) formula was generated by the sum of products of the gene expression level and its corresponding coefficients. Area under the curve (AUC) of the time-dependent receiver operating characteristic (ROC) curve and Harrell’s concordance index (C-index) were used to evaluate the prognostic value of the risk score model.

### TIMER

Tumor Immune Estimation Resource (TIMER^5^.) website is a reliable platform for comprehensively investigate molecular characterization of tumor-immune interactions based on transcript per million reads (TPM) from TCGA contains 10,897 tumors across 32 cancer types (Li et al., 2017). In our study, the Gene module was used to explore the correlation between six major tumor-infiltrating immune subsets and CXCL10 expression.

### Statistical Analysis

Statistical analysis was performed with R package (version 3.6.1,^6^ and GraphPad Prism version 6 (GraphPad Software, La Jolla, CA, United States). Categorical variables were analyzed with Pearson’s chi-square test, while continuous variables were compared with Student’s *t*-test or the Wilcoxon rank sum test for two groups and one-way ANOVA for multiple groups. The Kaplan-Meier survival curve was generated to illustrate the correlation between differential factors and overall survival, and the log-rank test was used to assess the significant difference between the groups. Univariate and multivariate Cox regression analyses were performed by the R survival package. Time-dependent ROC curve was generated with the TimeROC package, and the C-index was calculated and analyzed with the survcomp package. Unless otherwise mentioned, a two-sided *p*-value of < 0.05 was considered to be statistically significant.

## Results

### Association of Immune/Stromal Scores With Pancreatic Cancer Pathology and Prognosis

**FIGURE 1:**
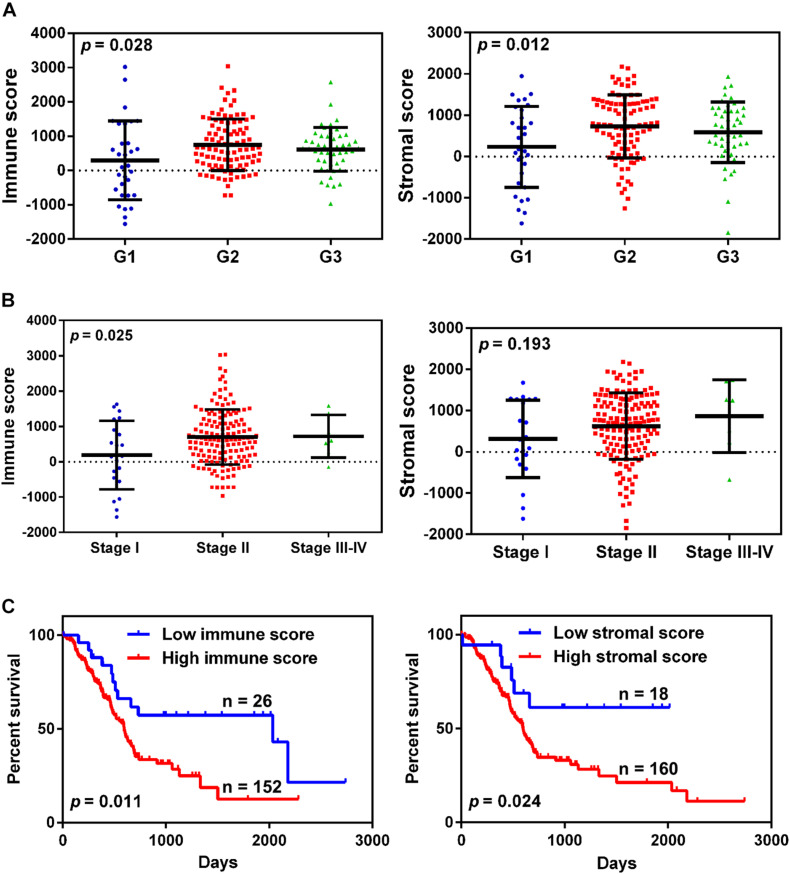
The relationship between immune/stromal scores and pancreatic cancer clinical pathological features. **(A,B)** Distribution of immune/stromal scores in tumor grades **(A)** and TNM stage **(B)**. **(C)** Kaplan-Meier survival curves for overall survival in the high and low immune/stromal score groups.

A total of 178 patients with complete gene expression profiles and clinical information were included in our study from the TCGA database. To determine the role of immune/stromal scores in the prognosis of pancreatic cancer, we calculated the immune/stromal scores using the ESTIMATE algorithm. The immune and stromal scores ranged from −1,559.87 to 3,037.78 and from −1,843.32 to 2,179.19, respectively. The association of the immune/stromal scores with pancreatic cancer pathology was investigated by comparing the score distributions among tumor differentiation grades and TNM stages. Both immune and stromal scores roughly increased with poor tumor differentiation grades (one-way ANOVA test, *p*-value = 0.028 and 0.012 for immune and stromal scores, respectively, Figure 1A). A significant relationship was observed between immune scores and TNM stages (one-way ANOVA, *p*-value = 0.025, Figure 1B), and a similar result was also observed in T stages (*t*-test, *p*-value = 0.043, Supplementary Figure 1A), whereas the stromal scores did not show a significant relationship with TNM stages. In addition, patients with lymph node metastasis had higher immune/stromal scores, although it was not statistically significant (Supplementary Figure 1B).

To determine the correlation between the immune/stromal scores and overall survival, the patients were classified into low and high score groups by using X-tile software. The Kaplan-Meier survival curve indicated that the median overall survival of patients with high immune scores was shorter than that of patients with low scores (439 vs. 855 days, *p*-value = 0.011, Figure 1C). Simultaneously, increased stromal scores predicted shorter overall survival for patients with pancreatic cancer (447 vs. 632 days, *p*-value = 0.024, Figure 1C).

### Identification of DEGs Based on the Immune/Stromal Scores

**FIGURE 2:**
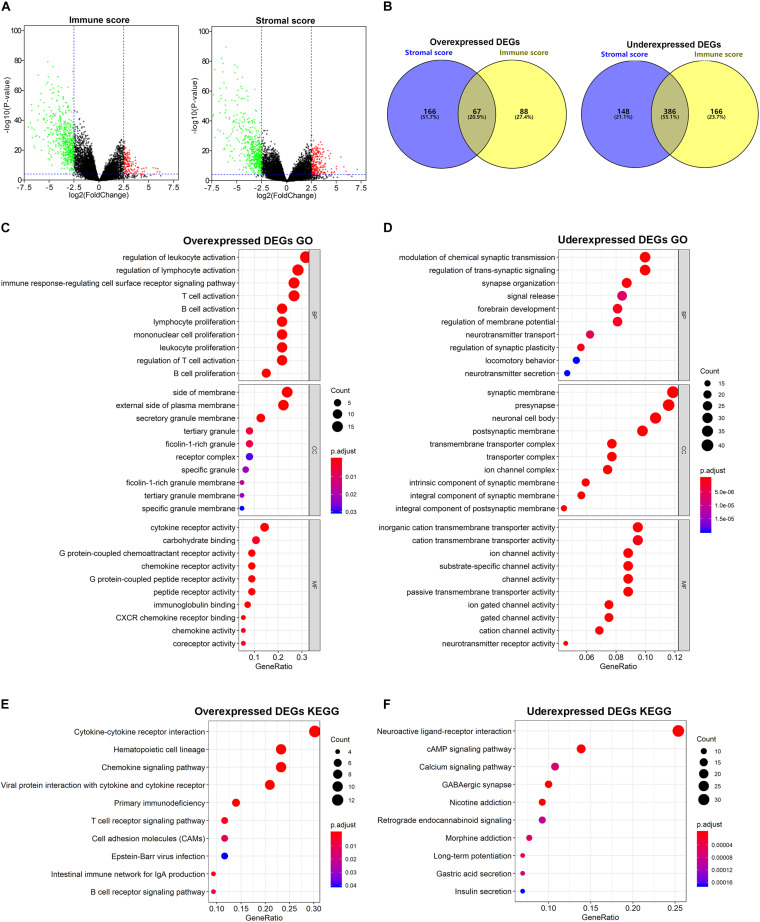
Comparison of gene expression profile with immune/stromal scores. **(A)** Volcano plots of DEGs from the low vs. high immune/stromal score groups. |Log_2_FC (fold change)| > 2.5, FDR adjusted *p*-value < 0.001. **(B)** Commonly changed DEGs in immune/stromal score groups. **(C,D)** The top 10 GO terms for commonly changed DEGs. **(E,F)** The top 10 KEGG enrichment pathways for commonly changed DEGs.

To determine DEGs based on the immune/stromal scores, gene expression profiles from 178 TCGA cases were analyzed. We identified 707 genes to be immune score-related DEGs, among which 155 genes were overexpressed and 552 genes were underexpressed. Regarding stromal score-related DEGs, 767 genes were ascertained to be related to stromal scores, including 233 overexpressed genes and 534 underexpressed genes. These immune/stromal score-related DEGs were visualized on the volcano plots (Figure 2A). Venn plots showed 67 DEGs that were commonly overexpressed in both the immune and stromal score groups, while 386 DEGs were found to be commonly underexpressed (Figure 2B). Subsequently, we further analyzed these intersected DEGs.

### GO Term and KEGG Pathway Enrichment Analyses of the Intersected DEGs

To investigate the potential biological function of the intersected DEGs, we conducted GO and KEGG pathway enrichment analyses. According to GO enrichment results, the overexpressed DEGs were significantly enriched in inflammatory cell activation and proliferation, and cytokine and chemokine receptor activity, while the underexpressed DEGs were mainly involved in transmembrane transporter activity and channel activity. The top 10 function annotations for the overexpressed and underexpressed DEGs are shown in Figures 2C,D, respectively. Additionally, in the enrichment analysis of KEGG pathways, the overexpressed DEGs were significantly associated with inflammation cell pathways and cytokine receptor interaction, while the underexpressed DEGs were mostly observed in secondary messenger-related and drug-related signaling pathways. The top 10 pathways selected for the overexpressed and underexpressed DEGs in the enrichment analysis are shown in Figures 2E,F, respectively.

### Protein-Protein Interaction Network Analysis

To further determine the interactions among the 67 commonly overexpressed DEGs, protein-protein interaction (PPI) networks were constructed using the STRING database and Cytoscape software. As shown in Supplementary Figure 2A, the PPI network comprised 58 nodes and 323 edges. We then performed clustering analysis of the PPI network by using Cytotype MCODE, and the modules that included at least 10 nodes were selected. As shown in Supplementary Figure 2B, module 1 contained 13 nodes and 61 edges. The important hub genes were associated with chemokines and their receptors, such as CXCL9, CXCL10, CXCL13, CCR7, CXCR5, and CXCR2. In addition, module 2 contained 14 nodes and 51 edges, and the vital genes included CD19, PTPRC, CD79B, IL21R, TNFRSF13C, and MS4A1 (Supplementary Figure 2C).

### GSEA

**FIGURE 3:**
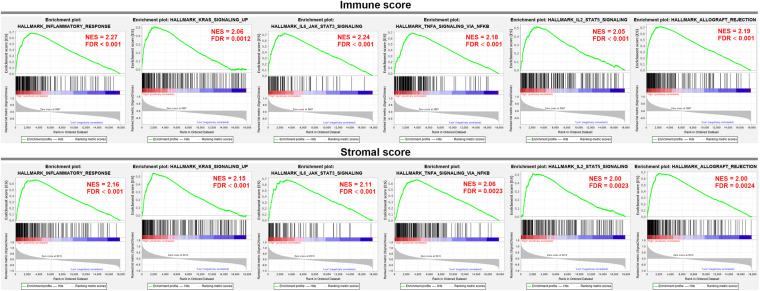
GSEA analysis was applied to identify the significant hallmark gene sets in the high immune/stromal score group.

To elucidate the biological states or processes of the immune/stromal scores, GSEA was conducted to search hallmark gene sets. In the high immune score group, eight gene sets were significantly enriched based on the criteria of |NES| ≥ 2 and FDR adjusted *p*-value < 0.01, while nine gene sets were enriched in the high stromal score group. Interestingly, they shared the six gene sets, including INFLAMMATORY_RESPONSE, KRAS_SIGNALING_UP, IL6_JAK_STAT3_SIGNALING, TNFA_SIGNALING_VIA_NFKB, IL2_STAT5_SIGNALING, and ALLOGRAFT_REJECTION (Figure 3), which were associated with the regulation of the inflammatory process.

### Construction and Validation of a Prognostic Risk Model

**FIGURE 4:**
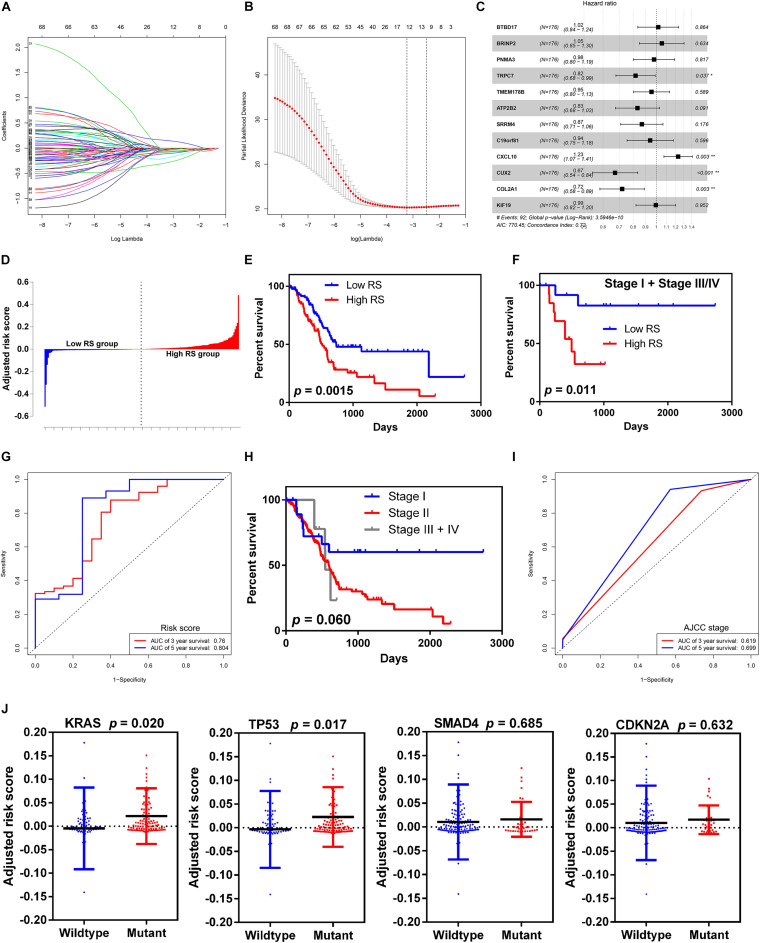
Selection of microenvironment related DEGs and construction the risk score model. **(A)** Trend graph of LASSO coefficients. **(B)** Partial likelihood deviation map. **(C)** Forest plot of hazard ratios showing the results of multivariate Cox regression analysis. The four genes (TRPC7, CXCL10, CUX2, and COL2A1) were identified as the independent risk factors. **(D)** Distribution of adjusted risk scores. **(E)** Kaplan-Meier survival curves for overall survival in the high and low risk score groups in all stages. **(F)** Kaplan-Meier survival curves for overall survival in the high and low risk score groups in stage I and stage III/IV. **(G)** Time-dependent ROC analysis of risk score model. **(H)** Kaplan-Meier survival curves for overall survival in different AJCC stage. **(I)** Time-dependent ROC analysis of AJCC stage. **(J)** Distribution of risk scores for the four genes (KRAS, TP53, SMAD4, and CDKN2A) with mutant or wildtype status.

To screen the prognostic significance of the identified DEGs described above, the univariate Cox regression analysis was performed. We chose 68 of the 453 intersected DEGs with *p*-value < 0.001 for further analysis. The LASSO regression model was then used to discriminate key prognosis-related genes by using the R package glmnet. After calculation and verification, the results showed that the model consisted of 12 genes with the minimum partial likelihood deviance (Figures 4A,B), which included BTB domain containing 17 (BTBD17), BMP/retinoic acid-inducible neural specific 2 (BRINP2), PNMA family member 3 (PNMA3), transient receptor potential cation channel subfamily C member 7 (TRPC7), transmembrane protein 178B (TMEM178B), ATPase plasma membrane Ca^2+^ transporting 2 (ATP2B2), serine/arginine repetitive matrix 4 (SRRM4), chromosome 19 open reading frame 81 (C19orf81), C-X-C motif chemokine ligand 10 (CXCL10), cut-like homeobox 2 (CUX2), collagen type II alpha 1 chain (COL2A1), and kinesin family member 19 (KIF19). Lastly, these 12 genes were fit into the multivariate Cox regression analysis model to determine the independent risk genes. The results showed that four genes, including TRPC7 (HR 0.82, *p*-value = 0.037), CXCL10 (HR 1.23, *p*-value = 0.003), CUX2 (HR 0.67, *p*-value < 0.001), and COL2A1 (HR 0.72, *p*-value = 0.003), were identified as independent prognostic indicators (Figure 4C). Therefore, we constructed a prognostic risk model by computing the sum of the products of the expression level of the abovementioned four genes and their corresponding coefficients. The risk score (RS) formula was as follows: (−0.1998 × expression level of TRPC7) + (0.2074 × expression level of CXCL10) + (−0.3961 × expression level of CUX2) + (−0.3281 × expression level of COL2A1).

We grouped the 178 patients with complete clinical information from the TCGA dataset into the high-RS group and the low RS group according to the median score (Figure 4D). The high RS group showed a higher frequency of poor overall survival than the low RS group (417.5 vs. 504.5 days, *p*-value = 0.0015, Figure 4E). Moreover, we validated the risk score model in stage I and stage III/IV, because the majority of patients with pancreatic cancer in the TCGA cohort were in stage II. Similarly to the entire cohort, patients with high RS had significantly poorer overall survival than those with low RS (424 vs. 859 days, *p*-value = 0.011, Figure 4F). Time-dependent ROC and C-index were used to evaluate the prognostic value of the risk score model in comparison with the AJCC stage (Figures 4G–I). The AUCs of the risk score model at 3- and 5-year overall survival were 0.760 and 0.804, respectively, and both were superior compared to that of the AJCC stage (0.619 and 0.699 for 3- and 5-year, respectively). The risk score model [95% confidence interval (CI), 0.652 to 0.770] showed higher C-index than the AJCC stage (95% CI, 0.468 to 0.550).

To determine the relationship between the risk score level and gene mutation status in pancreatic cancer, we selected the four leading mutation genes (KRAS, TP53, SMAD4, and CDKN2A) for further analysis. The results demonstrated that patients with KRAS and TP53 mutant genes had significantly higher risk score level than those with wild-type genes (*p*-value = 0.020 and 0.017 for KRAS and TP53, respectively) (Figure 4J). However, no significant difference was found in SMAD4 and CDKN2A mutations.

### Identification of CXCL10 as an Important Chemokine in Pancreatic Cancer Microenvironment

**FIGURE 5:**
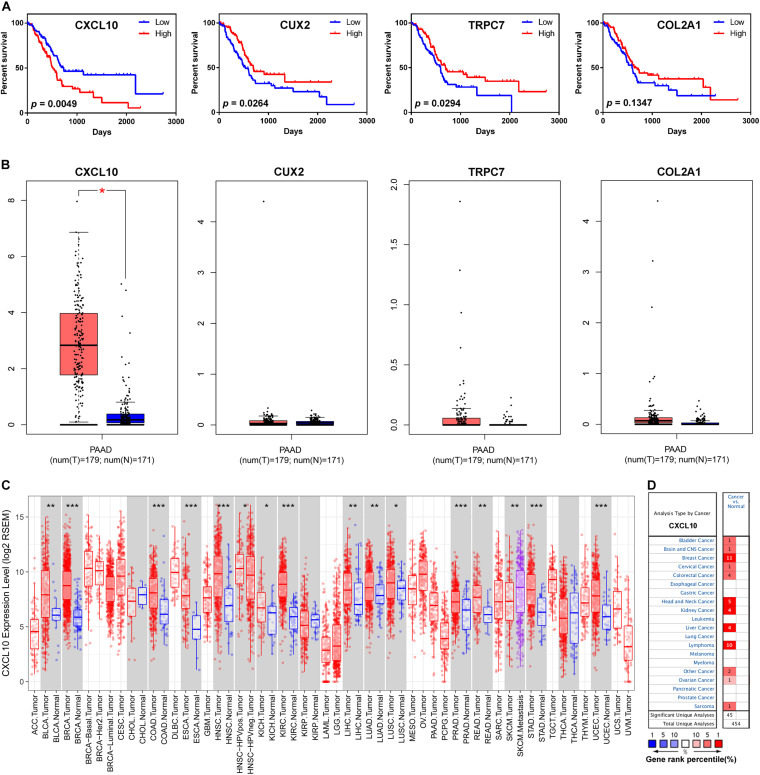
CXCL10 expression levels in human cancers. **(A)** Kaplan-Meier survival curves for overall survival based on the four gene expression levels. **(B)** The four gene expression levels in pancreatic cancer (from TCGA database) and normal pancreas (from GTEx database) were analyzed by GEPIA online website. **(C)** CXCL10 expression levels in different cancers (from TCGA database) were determined by TIMER website. *p-value < 0.05, **p-value < 0.01, and ***p-value < 0.001. **(D)** CXCL10 expression levels in data sets of different cancers in the Oncomine database. The four genes: TRPC7, CXCL10, CUX2, and COL2A1.

To confirm the potential roles of each gene from the risk score model in overall survival, we performed the Kaplan-Meier survival analysis. High expression level of CXCL10 was negatively associated with overall survival, while high expression level of CUX2 and TRPC7 was positively correlated with overall survival; no significant difference was observed for COL2A1 (Figure 5A). Subsequently, the expression levels of the four genes were validated through the Gene Expression Profiling Interactive Analysis (GEPIA) online website^7^ (Tang et al., 2017). Only the mRNA expression level of CXCL10 was remarkably increased in pancreatic cancer tissue compared to that in normal pancreatic tissues from the Genotype-Tissue Expression (GTEx) project (Figure 5B); this project was considered for comparison because there are few normal pancreatic tissues in the TCGA database. Furthermore, we investigated the expression level of CXCL10 in other solid tumors by using the TIMER online website^8^ (Li et al., 2017) and the Oncomine database^9^ (Rhodes et al., 2004). The results showed that the expression level of CXCL10 was upregulated in most solid tumors, including bladder cancer, breast cancer, colorectal cancer, head and neck cancer, and liver cancer (Figures 5C,D). We also found that the metastatic lesions of skin cutaneous melanoma had higher expression level of CXCL10 than the primary lesions (Figure 5C).

**FIGURE 6:**
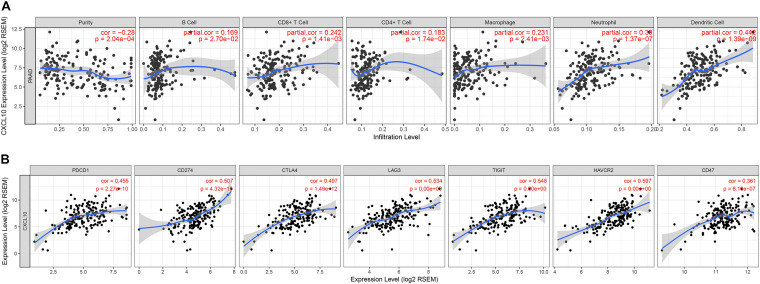
Correlation between the expression of CXCL10 and the infiltration of immune cells **(A)** or the expression of immune checkpoints **(B)**, were analyzed by TIMER website.

To determine the correlation between CXCL10 and tumor-infiltrated immune cells, we performed a correlation analysis by using the TIMER online website. TIMER website is a comprehensive resource which allow us to explore the correlation between six major tumor-infiltrating immune subsets (B cells, CD4 + T cells, CD8 + T cells, Neutrophils, Macrophages, and Dendritic cells) and gene expression. The results showed that the expression level of CXCL10 positively correlated with macrophages, CD8^+^ T cells, neutrophils, and dendritic cells (Figure 6A). In contrast, it negatively correlated with tumor purity. Moreover, the expression level of CXCL10 showed a significant positive correlation with the immune checkpoint-related proteins such as PCCD1, CD274, CTLA4, LAG3, TIGIT, and HAVCR2 (Figure 6B).

**FIGURE 7:**
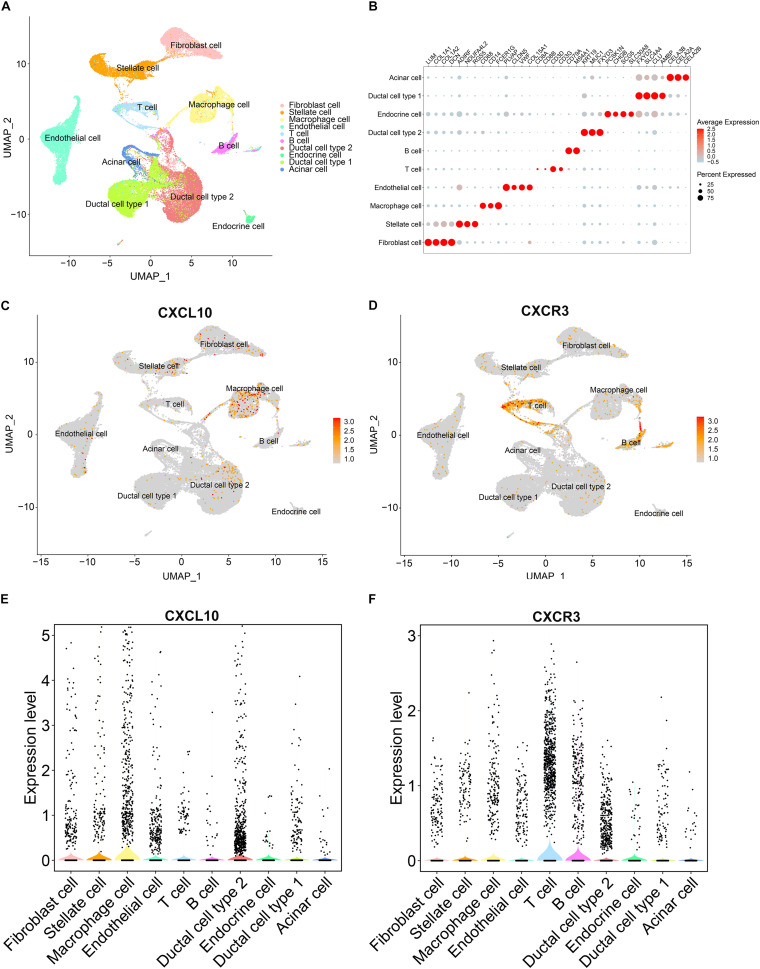
The gene expression analysis from published scRNA-seq profiling of pancreatic cancer. **(A)** Depiction of UMAP dimensional reduction. Ten major cell types were identified. **(B)** Dotplot for expression of key lineage marker genes across the ten cell types. **(C,D)** Expression levels of CXCL10 and CXCR3 for all identified cell types were visualized on the UMAP plot. **(E,F)** Violin plots of CXCL10 and CXCR3 expression in all identified cell types. Black dots showed the expression level of CXCL10 or CXCR3 for each cell.

To identified the main source of CXCL10, we downloaded an available human pancreatic cancer single-cell RNA-sequencing (scRNA-seq) data (Peng et al., 2019) from the Genome Sequence Archive in the BIG Data Center, Chinese Academy of Sciences under accession code CRA001160^10^ (Members, 2018). Uniform Manifold Approximation and Projection (UMAP) and key lineage marker gene analyses were carried out for cell type identification (Figures 7A,B). Then we analyzed the RNA expression of CXCL10 and its receptor CXCR3 at single-cell level in pancreatic cancer. Both UMAP plot and violin plot demonstrated that tumor associated macrophages were more likely the main source of CXCL10 and its receptor CXCR3 was highly expressed in T cells (Figures 7C–F). These findings were largely consistent with House’ findings (House et al., 2019) that macrophage secreted abundant CXCL10 and CXCL9 to promote the infiltration of CD8 + T cell in pancreatic cancer following immune checkpoint blockade.

## Discussion

A pathological characteristic of pancreatic cancer is the dense desmoplastic reaction in the tumor stroma, resulting in shaping of the unique TME. Several studies have proven that TME is involved in pancreatic cancer initiation, progression, and response to therapies, especially tumor immunotherapy. Immune and stromal cells are the major non-tumor cells in the TME, and they may provide new perspectives in cancer research. In the present study, we attempted to determine the infiltration level of immune/stromal cells in pancreatic cancer by calculating their scores with the ESTIMATE algorithm. The Kaplan-Meier survival analysis revealed that both low immune scores and low stromal scores predicted a favorable prognosis in patients with pancreatic cancer. In addition, the immune scores were associated with differentiation grades and TNM stage, while high stromal scores were also related to poor differentiation.

To investigate the potential mechanism of the difference in immune/stromal scores, we identified DEGs between the high and low score groups used for the functional enrichment analysis. A total of 453 overlapping DEGs, including 67 overexpressed and 386 underexpressed genes, were determined. The GO term analysis showed that these DEGs were mainly enriched in the cytokine receptor activity, chemokine receptor activity, G protein-coupled receptor activity, and transmembrane transporter activity. The KEGG pathway enrichment analysis showed that the DEGs were predominantly clustered in the cytokine-cytokine receptor interaction pathway, chemokine signaling pathway, and T/B cell receptor signaling pathway. Cytokines and chemokines are important inflammatory mediators in TME, and they are involved in regulating tumor formation and progression through binding to specific receptors on the surface of target cells (Atretkhany et al., 2016). The GSEA enrichment analysis showed that the high immune and stromal score group shared six gene sets including IL6 and TNF-α signaling, which were closely related to the inflammatory process. IL6 *trans-*signaling promotes pancreatic intraepithelial neoplasia to progress to pancreatic cancer (Lesina et al., 2011) and drives niche formation in liver metastasis (Thomas, 2019). In contrast, the inhibition of IL6 signaling reduces pancreatic cancer growth and recurrence in xenograft models (Goumas et al., 2015). Furthermore, IL6 antibody blockade enhances the efficacy of anti-PD-L1 therapy in patients with pancreatic cancer (Mace et al., 2018). TNF-α has been shown to increase the antitumor activity of gemcitabine in pancreatic cancer (Murugesan et al., 2009); however, its curative effect does not seem to be perfect due to the activation of the NF-κB signaling pathway (Egberts et al., 2008). Hence, Li et al. (2010) used maslinic acid to potentiate the antitumor activity of TNF-α by suppressing NF-KB activation. In addition, a recent study demonstrates that activated macrophage-derived TNF-α can upregulate PD-L1 expression in pancreatic cancer cells, which leads to poor prognosis (Tsukamoto et al., 2019).

To identify the independent prognostic TME-related genes, we used the LASSO and multivariate Cox regression analyses to screen the gene signatures among the 453 DEGs. We then constructed a risk score prediction model consisting of TRPC7, CXCL10, CUX2, and COL2A1. The survival analysis showed that the high RS group was correlated with poor overall survival. Compared with AJCC stage, the AUC and C-index confirmed that the risk score model was superior in predicting 3- and 5-year overall survival; this finding suggested that the risk score model could improve the prediction accuracy of AJCC stage in pancreatic cancer. A previous study reported that the combination of immune scores in TME and AJCC stage had better prognostic value than AJCC stage alone in patients with gastric cancer (Jiang et al., 2018). Moreover, we found a significant increase in risk scores in patients with KRAS and TP53 mutation. KRAS is the most frequently mutated oncogene in pancreatic cancer, and recent studies have verified that the activation of KRAS in cancer cells affects the properties and functions of the surrounding microenvironment (Dias Carvalho et al., 2018). The TP53 mutation was also found to be strongly associated with the immune microenvironment in hepatocellular carcinoma (Long et al., 2019), and it can significantly enhance the expression of immune checkpoint-related proteins and serve as a predictor of immunotherapy in lung cancer (Dong et al., 2017).

Notably, among the four genes signature, only the CXCL10 gene was involved in tumor immunity; however, few studies have been conducted in oncology for the other three genes (TRPC7, CUX2, and COL2A1). Hence, further research on these three genes is needed, particularly in pancreatic cancer. We then focused our attention on CXCL10. The expression level of CXCL10 in pancreatic cancer tissue was higher than that in normal pancreatic tissue, and high expression level of CXCL10 was correlated with shorter overall survival. In addition, both TIMER online website and the Oncomine database confirmed the correlation of the high expression level of CXCL10 with many malignancies. Because CXCL10 is involved in regulating immune cell migration, differentiation, and activation, we examined the correlation between CXCL10 and immune cells in TIMER by using Spearman’s correlation analysis. The results showed that CXCL10 was negatively correlated with tumor purity; this finding suggested that the main source of CXCL10 was stromal cells but not cancer cells. Lunardi et al. (2015) showed that CXCL10 is highly expressed by pancreatic stellate cells in the presence of pancreatic cancer cells, and its expression is associated with the infiltration of regulatory T cells (Tregs) and poor overall survival. In our present study, we found that the expression level of CXCL10 correlated with the infiltration of various immune cells, especially dendritic cells. CD103^+^ dendritic cells can secrete abundant CXCL9/10 to recruit effector T cells into the TME to repress tumor immune escape in melanoma (Spranger et al., 2017). However, we found that CXCL10 was predominantly expressed by tumor associated macrophages and its receptor CXCR3 was highly expressed in T cells at the single-cell level in pancreatic cancer. It seems that CXCL10 exerted different effects in different malignant tumors and played an important role in TME. CXCL10 promotes tumor growth and metastasis in colon cancer (Zipin-Roitman et al., 2007; Wightman et al., 2015) and liver cancer (Ling et al., 2014; Li et al., 2016); however, it inhibits melanoma invasiveness and lung metastasis (Mirzaei et al., 2018) and improves therapeutic effect in breast cancer (House et al., 2020). We also found that the expression level of CXCL10 was positively correlated with several immune checkpoint-related proteins, which implied that CXCL10 could be used as an effective predictor of immunotherapy. Recently, (House et al. (2020) reported that CXCL9/10 is predominantly expressed by macrophages after dual PD-1/CTLA4 blockade to enhance patient response. In short, a better understanding of CXCL10 in TME is required to understand its role in tumor progression and treatment.

Our present study had a major limitation. Because all cases were selected from the TCGA public database, the underlying selection bias could not be avoided; therefore, the TME-related prognostic genes should be further validated to determine their regulatory mechanism. To exclude the bias, we intend to test their effectiveness in clinical specimens and conduct further *in vitro* and *in vivo* research to testify the findings of this study.

## Conclusion

In conclusion, an integrated bioinformatics analysis of pancreatic cancer dataset from the TCGA was performed using the ESTIMATE algorithm, and we constructed a risk score model comprising four DEGs to provide a better method for predicting survival condition than AJCC stage. Further studies on these genes are required to gain further insight into their molecular mechanism in pancreatic cancer initiation and progression.

## Data Availability Statement

The TCGA data used and analyzed in this study are available from UCSC Xena (https://xena.ucsc.edu/), and the single-cell RNA-seq data were downloaded from the Genome Sequence Archive in the BIG Data Center, Chinese Academy of Sciences under accession code CRA001160 (https://bigd.big.ac.cn/bioproject/browse/PRJCA001063).

## Author Contributions

SC, GL, and KH: conception and design. FH, SC, and GL: data collection. YC, JL, and YL: data analysis and interpretation. SC and KH: manuscript writing. All authors have approved the final manuscript.

## Conflict of Interest

The authors declare that the research was conducted in the absence of any commercial or financial relationships that could be construed as a potential conflict of interest.

## Footnotes

1 https://xena.ucsc.edu/

2 https://bioinformatics.mdanderson.org/estimate/

3 https://www.cbioportal.org/

4 https://bioinfogp.cnb.csic.es/tools/venny/index.html

5 https://cistrome.shinyapps.io/timer/

6 https://www.r-project.org/

7 http://gepia.cancer-pku.cn/

8 https://cistrome.shinyapps.io/timer/

9 https://software.oncomine.com/resource/main.html

10 https://bigd.big.ac.cn/bioproject/browse/PRJCA001063
