# Supplementary material for: Bioinformatics-Based Identification of Tumor Microenvironment-Related Prognostic Genes in Pancreatic Cancer
Source: Front Genet. 2021 Jun 30;12:632803. doi: 10.3389/fgene.2021.632803 (PMC8277941; doi:10.3389/fgene.2021.632803)
Supplement: Supplementary file 2 [file Data_Sheet_2.docx]

**Supplementary Data**

**Protein-Protein Interaction (PPI) Network**

The Overexpressed DEGs were imported into STRING database with minimum required interaction score more than 0.4 to construct PPI network(Szklarczyk et al., 2019). The data of nodes from the PPI network was further analyzed by Cytoscape software (version 3.7.2)(Shannon et al., 2003). In addition, Molecular Complex Detection (MCODE) (Bader and Hogue, 2003)was used to find densely connected regions by selecting clusters with node numbers greater than 10.

**Supplementary Data 1.** A gene list of immune and stromal signatures.

**Supplementary Figure 1.** Distribution of immune/stromal scores in T stage (A) and lymph node status (B).

**Supplementary Figure 2.** PPI network of DEGs and module identification. (A) The PPI network of 67 overexpressed DEGs. (B-C) Module 1 (B) and module 2 (C) were the top two modules (> 10 nodes) in the PPI network (A). The color of nodes indicated the combine scores.

**References**

Bader, G.D., and Hogue, C.W. (2003). An automated method for finding molecular complexes in large protein interaction networks. *BMC Bioinformatics* 4**,** 2. doi: 10.1186/1471-2105-4-2.

Shannon, P., Markiel, A., Ozier, O., Baliga, N.S., Wang, J.T., Ramage, D., et al. (2003). Cytoscape: a software environment for integrated models of biomolecular interaction networks. *Genome Res* 13(11)**,** 2498-2504. doi: 10.1101/gr.1239303.

Szklarczyk, D., Gable, A.L., Lyon, D., Junge, A., Wyder, S., Huerta-Cepas, J., et al. supporting functional discovery in genome-wide experimental datasets. *Nucleic Acids Res* 47(D1)**,** D607-d613. doi: 10.1093/nar/gky1131.
