# Supplementary figures and images for: Bioinformatics-Based Identification of Tumor Microenvironment-Related Prognostic Genes in Pancreatic Cancer
Source: Front Genet. 2021 Jun 30;12:632803. doi: 10.3389/fgene.2021.632803 (PMC8277941; doi:10.3389/fgene.2021.632803)

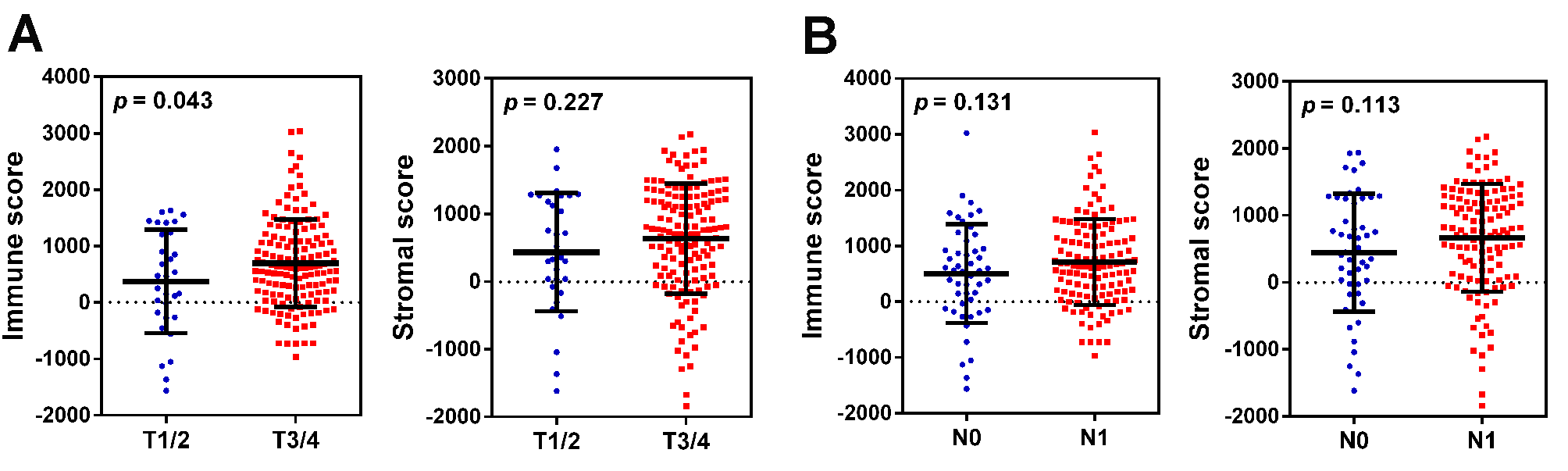

Supplement: Supplementary file 3 [file Image_1.TIF]

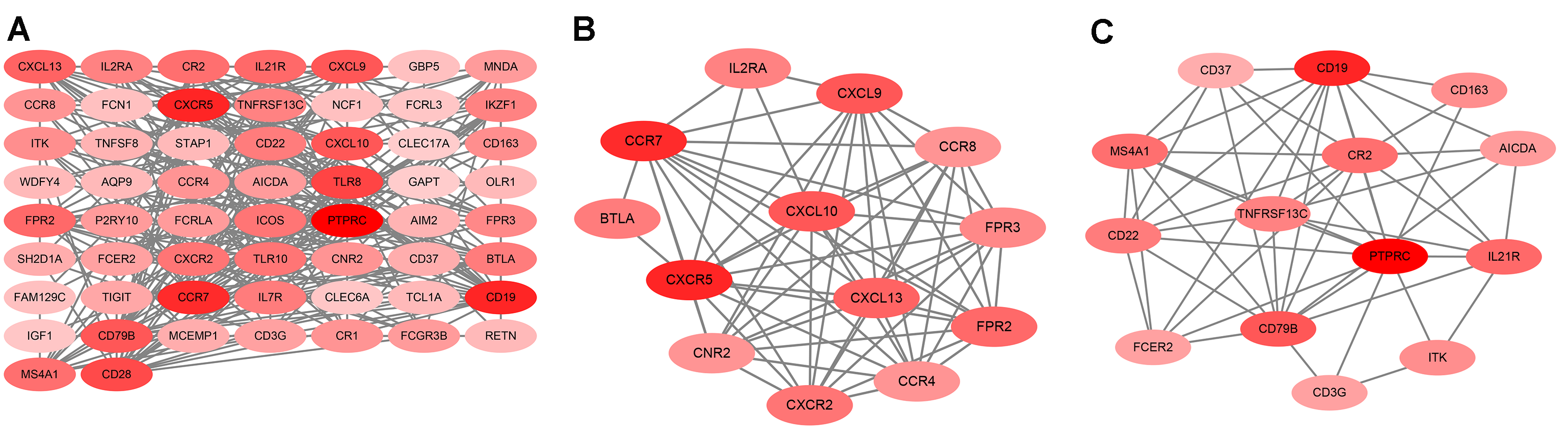

Supplement: Supplementary file 4 [file Image_2.TIF]
